# Functional Outcomes of Patients With Proximal Humerus Fractures Treated With Primary Versus Salvage Reverse Shoulder Arthroplasty: A Cohort Study of 41 Patients

**DOI:** 10.7759/cureus.83460

**Published:** 2025-05-04

**Authors:** Antonio Ariztía, Andrés Calvo, Vicente Alba, Daniel Paccot, Nazira Bernal, Cristian Aravena, Felipe Reinares

**Affiliations:** 1 Orthopaedics and Traumatology, Facultad de Medicina Clinica Alemana Universidad del Desarrollo, Santiago, CHL; 2 Orthopaedics and Traumatology, Hospital Clinico Mutual de Seguridad Cámara Chilena de la Construcción (CChC), Santiago, CHL; 3 Orthopaedic Surgery, Facultad de Medicina Clinica Alemana Universidad del Desarrollo, Santiago, CHL

**Keywords:** failed treatment, fracture sequelae, humerus, proximal humerus, proximal humerus fracture, replacement, reverse shoulder arthroplasty

## Abstract

Introduction

Reverse shoulder arthroplasty (RSA) is an option for patients with complex fractures of the proximal humerus and a salvage option for osteosynthesis failure, fracture sequelae, and hemiarthroplasty (HA) revision. The objective of this study was to compare the functional outcomes of primary RSA for proximal humerus fractures (PHFs) with those of salvage RSA for the failed treatment of PHFs. We hypothesized that patients undergoing primary RSA would experience significantly less pain and better functional outcomes than those receiving RSA as a salvage procedure.

Methods

This was a retrospective cohort study of patients with PHFs who underwent RSA initially or as a salvage procedure due to failure of conservative treatment, osteosynthesis, or HA. The demographic characteristics of the patients and the radiological features of the initial fracture were considered. Functional outcomes were assessed in terms of range of motion (ROM), the Constant-Murley score (CMS), the subjective shoulder value (SSV), and pain (visual analog scale (VAS)). For the comparative statistical analysis, the t-test and Mann-Whitney U test were used.

Results

Ninety patients underwent RSA, but only 41 patients were included. There were 23 patients in the primary RSA group and 18 in the salvage RSA group, with an average follow-up of 15 months (12-80 months). The primary RSA group experienced significantly less pain (VAS: 0.5±0.13 vs. 1.8±0.40) (p=0.002) and had a better CMS functional index score (73.2±1.6 vs. 65±3.01) (p=0.01) at the end of the follow-up period. No differences in ROM (active anterior elevation and external or internal rotation) or SSV were observed between the two groups.

Conclusions

In this series of patients, ROM was similar among the patients who underwent primary RSA and those who underwent salvage RSA; however, those who underwent primary RSA had better but not clinically relevant CMS and significantly less pain.

## Introduction

Proximal humerus fractures (PHFs) have increased in frequency due to population aging, with a threefold rise estimated over the past 30 years [1]. Currently, PHFs are the second most common fracture of the upper limb after distal radius fractures and are associated with low bone mineral density [1].

Common treatment options for PHFs include open reduction and internal fixation (ORIF), hemiarthroplasty (HA), reverse shoulder arthroplasty (RSA), or conservative management. While most PHFs can be managed nonsurgically [2], the surgical choice for complex displaced PHFs remains controversial [2-4].

RSA has been used as an acute treatment option for displaced PHFs in elderly individuals and as a salvage option for failed ORIF, failed HA, or fracture sequelae [5-11]. While some studies revealed that primary RSA leads to better functional results and ROM [12-15], others have revealed similar results, suggesting that nonsurgical treatment should be performed first and RSA should be performed as a salvage procedure [12,16]. Despite the clinical results, a higher complication rate and lower tuberosity healing rate have been reported with salvage RSA [12-15,17,18].

The aim of this study is to compare primary RSA with salvage RSA in terms of functional outcomes and ROM after PHF.

The present study was previously presented as a free paper at the International Congress on Shoulder and Elbow Surgery (ICSES) in Rome on September 5-8, 2023.

## Materials and methods

A retrospective cohort study was conducted between January 2015 and July 2022. We included all patients with PHFs who were treated with RSA by the senior author at a single level I trauma center, Hospital Clinico Mutual de Seguridad, located in the Metropolitan Region of Santiago, Chile. Patients with open PHFs, infections, prior humerus or scapula fractures, ipsilateral upper extremity fractures, or neurological injuries of the same arm were excluded. Patients were categorized into two groups: primary RSA and salvage RSA.

The primary RSA group included patients with irreparable fractures resulting from calcar comminution, head-split fractures, and proximal head fracture dislocations and patients older than 70 years with greater tuberosity displacement. The salvage RSA group included patients whose nonoperative treatments, ORIF, or HA failed. Failures were characterized by avascular necrosis (AVN), aseptic loosening of the implants, tuberosity malunion, limited range of motion (ROM), or complications following ORIF, such as varus collapse, screw cut-out, or articular screw penetration.

The active range of motion (AROM) of the shoulder, including forward elevation and external rotation at 0° of abduction, was assessed at the last follow-up. Internal rotation was assessed using the vertebral height method. Data were quantified numerically according to the Constant-Murley score (CMS) assignment [19]. Patient-reported outcome measures (PROMs) included the CMS [19] adjusted by sex and age, the subjective shoulder value (SSV) [19,20], and the visual analog scale (VAS) pain score. A physiotherapist who was not related to the patient's primary care recorded the AROM and PROMs in the patient's chart.

Surgical technique

Surgery was performed while the patients were in the beach chair position with general anesthesia and an interscalene block. The deltopectoral approach was used in all patients. In primary RSA, osteotomy of the lesser tuberosity is performed whenever it is intact. In salvage RSA, subscapular peeling was performed in all patients. One centimeter of the supraspinatus tendon was removed in all patients, keeping the infraspinatus attached if intact. The humerus component was distally cemented with a neck-shaft angle according to the implant used. A bone graft from the humeral head was used at the tuberosity-shaft interface. Greater tuberosity and lesser tuberosity fixation were performed in all patients who underwent primary RSA using four to six horizontal cerclage sutures around the stem, and no vertical sutures were used. In salvage RSA, the subscapular was not reattached in any patient.

Two different implant systems were used: the Comprehensive Shoulder System (Zimmer Biomet, Warsaw, IN, USA) and the Ascend Flex + Aequalis Reversed II (Stryker, Kalamazoo, MI, USA). The choice of implant depended on availability at the time of surgery. The glenosphere size was used according to the patient's needs, and the preoperative plan was created with 3D planning software.

Rehabilitation protocol

A standardized rehabilitation protocol was employed for all patients. A neutral rotation sling with 30° of abduction was used for four weeks. Self-assisted and physiotherapy-assisted passive ROM exercises started one day after the procedure, and physiotherapy-assisted ROM started two weeks after the procedure and continued until six weeks. At the sixth week, active ROM was started. Strengthening exercises were started at 12 weeks and were supervised by a physiotherapist for six months.

Radiological evaluation

The preoperative plan was created using CT scans. X-rays were taken from six to 12 months postoperatively and independently assessed by three trained shoulder orthopedic surgeons to assess the healing and/or resorption of tuberosities, and signs of radiolucency and implant loosening were evaluated according to the definitions of Melis et al. [21]. Tuberosity healing was considered complete if there was no evidence of displacement of the tuberosity, and trabeculation was noted between the humeral shaft and the greater tuberosity. In cases of disagreement, the case was reviewed with the senior author of the study, and a consensus was reached.

Statistical analysis

To compare the groups, nonparametric Mann-Whitney U tests and t-tests were used. A significance level of p<0.05 was established. Stata Statistical Software: Release 14.0 for macOS (April 2015; StataCorp LLC, College Station, TX, USA) was used for all the analyses.

Ethical approval for this study was obtained from the Ethics Committee of Hospital Clinico Mutual de Seguridad Cámara Chilena de la Construcción (CChC) (approval number: 2023-337) on July 6, 2023, in Santiago, Chile.

## Results

Forty-one patients met the study criteria, including 26 women and 15 men, with an average age of 64 years (range 47-80 years). Among them, 23 patients underwent primary RSA, and 18 patients underwent salvage RSA after failure of initial treatment.

**Figure 1:**
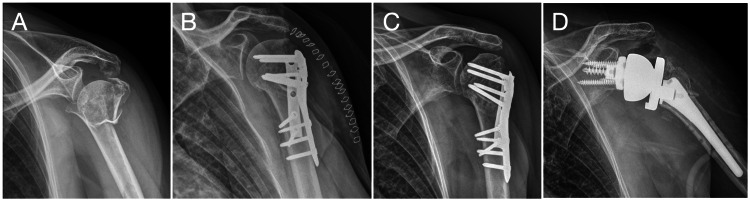
Case no. 5: salvage RSA case Left shoulder, man, 63 years old. (A) Four-part proximal humerus fracture, valgus impacted. (B) Initially treated with double plate (lateral anatomic locking plate and posterior 1/3 tube buttress plate). (C). Two years later developed AVN of the humeral head. (D) Salvage RSA. RSA: reverse shoulder arthroplasty; AVN: avascular necrosis

**Figure 2:**
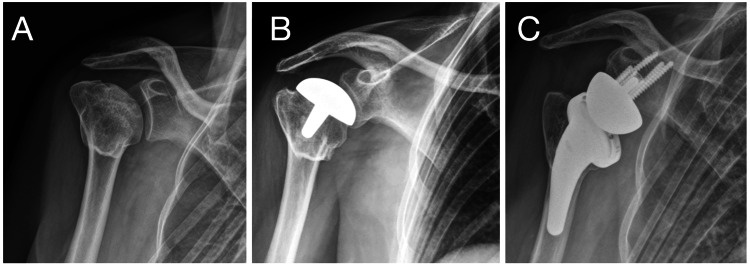
Case no. 14: salvage RSA case Right shoulder, woman, 60 years old. (A) Sequelae of proximal humeral head fracture, with greater tuberosity healed and AVN of the humeral head. (B) Initial treatment with humeral head resurfacing hemiarthroplasty. (C) Salvage RSA. RSA: reverse shoulder arthroplasty; AVN: avascular necrosis

In the salvage RSA group, 11 patients initially underwent osteosynthesis (Figure 1), five underwent HA, and two received nonsurgical treatment (Figure 2). The mean time between fracture and RSA in this group was 22 (SD±4) months. Table 1 shows the characteristics described above for each group.

**Table 1:** Characteristics for each group ORIF: open reduction and internal fixation; RSA: reverse shoulder arthroplasty

	Primary RSA	Salvage RSA
Men/women	26% (6)/74% (17)	50% (9)/50% (9)
Mean age (range)	67.9 (57-84)	62.9 (47-80)
Diabetes (%)	21.7	22.2
Tobacco (%)	8.7	5.5
Valgus impacted (%)	34.7	27.7
Varus impacted (%)	39	22.2
Neer classification		
2 parts	1	5
3 parts	8	6
4 parts	14	7
Initial treatment		
Conservative	0	2
ORIF	0	11
Hemiarthroplasty	0	5
RSA	23	0

**Figure 3:**
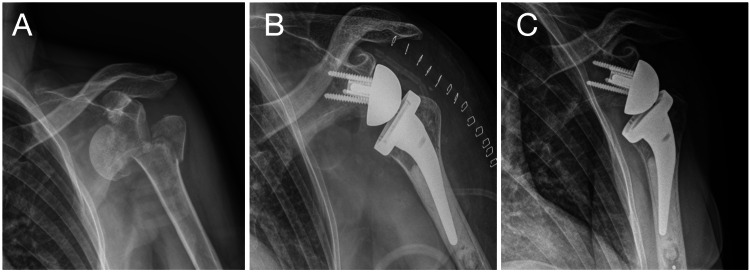
Case no. 38: primary RSA case Left shoulder, woman, 70 years old. (A) Three-part proximal humerus fracture with anterior dislocation. (B) Initial treatment with primary cemented RSA. (C) Follow-up at two years, complete healing of greater tuberosity. RSA: reverse shoulder arthroplasty

In the primary RSA group, there were 14 patients with four-part fractures, five patients with fracture dislocation, and three patients with head-split fractures (Figure 3).

Among all the fractures, 51.2% were four-part fractures, 34.2% were three-part fractures, and 14.6% were two-part fractures. Valgus impact was observed in 34% of the cases (n=14), whereas varus impact was noted in 32% (n=13). The average follow-up period was 27 months (ranging from 24 to 80 months).

In the salvage RSA group, among the patients who were initially treated with ORIF, six developed AVN (33.3%), and four experienced implant failure (17%). Among the patients who were treated with HA, the main cause of revision was tuberosity nonunion and poor function in three patients (16.7%). The cause for revision after each primary treatment in the salvage group is listed in Table 2.

**Table 2:** Cause for revision after each primary treatment in the salvage RSA group ORIF: open reduction and internal fixation; AVN: avascular necrosis; RSA: reverse shoulder arthroplasty

Primary treatment (18)	Cause of revision
ORIF (11)	6 AVN
	4 implant failure
	1 tuberosity nonunion
Hemiarthroplasty (5)	3 tuberosity nonunion
	1 tuberosity malunion
	1 aseptic loosening
Conservative (2)	1 tuberosity malunion
	1 surgical neck nonunion

At the final follow-up, the VAS score for pain in the primary RSA group was significantly lower, with an average of 0.52 (SD±0.66), than the score of 1.88 (SD±1.6) in the salvage RSA group (p=0.002). The mean anterior flexion and external rotation angles for the primary RSA group were 144° (SD±20) and 25° (SD±7), respectively. In the salvage RSA group, the ROM was 131° (SD±27) for anterior flexion and 26° (SD±8.9) for external rotation. There were no significant differences in ROM between the two groups (p=0.06 and p=0.4, respectively).

When comparing postoperative functional outcomes, the average CMS was 73 (SD±7) in the primary RSA group and 65 (SD±12) in the salvage group (p=0.01), and the difference was significant. The average SSV was 75 (SD±9) in the primary RSA group and 70 (SD±9) in the salvage group (p=0.06). The results are shown in Table 3.

**Table 3:** Comparison of postoperative functional outcomes between the groups Results are presented as percentage with standard deviation. ** represented with the arm at the side. ^✝^ represented in points as shown in the Constant-Murley score. VAS: visual analog scale; SSV: subjective shoulder value; RSA: reverse shoulder arthroplasty

	Primary RSA (n=23)	Salvage RSA (n=18)	P-value
VAS	0.52 (±0.66)	1.88 (±1.6)	0.002
Anterior flexion	144° (±20)	131° (±27)	0.06
External rotation**	25° (±7)	26° (±9)	0.4
Internal rotation^✝^	4 (±2)	4 (±2)	0.11
Constant-Murley score	73 (±7)	65 (±12)	0.01
SSV	75 (±9)	70 (±9)	0.06

Resorption of the tuberosities was observed in nine patients. In the primary RSA group, four cases of tuberosity resorption were observed, constituting 17% of all cases of tuberosity resorption, whereas in the salvage RSA group, five cases of resorption were observed, constituting 28% of all cases. There was no statistically significant difference between the groups, as evidenced by a p-value of 0.45.

There were no postoperative complications in either group.

## Discussion

The key finding of our study is that patients who underwent primary RSA for PHFs achieved superior functional outcomes and experienced significantly less pain compared to those who underwent salvage RSA, despite both groups having similar ROM. Specifically, the primary RSA group demonstrated a significantly higher CMS (73 vs. 65) and a significantly lower VAS pain score (0.5 vs. 1.8).

RSA can be used as an acute or salvage option after a PHF [5-11]. While some studies have revealed that primary RSA leads to better functional results and ROM [13-15,22], others have revealed similar results [12,16]. The results of our study indicate that the ROM was similar between patients who were treated with acute RSA and those who underwent salvage RSA after PHF. However, superior functional and pain scores were observed in the primary RSA group.

In our study, the primary RSA patients had a significantly higher CMS (73 vs. 65) and a significantly lower VAS pain score (1.8 vs. 0.5) than the salvage RSA patients did. Nevertheless, when considering a minimal clinically important difference (MCID) threshold of 5.7 for the CMS and 1.6 for the VAS pain score [23], only the CMS was clinically relevant.

There was a trend toward better forward elevation in the primary RSA group, with both groups having similar external rotation degrees. The functional ROM for activities of daily living is typically set at 120° for shoulder anterior flexion and 60° for external rotation [24]. Both groups achieved functional ROMs for anterior flexion but not for external rotation.

Seidl et al. [15] compared early versus late RSA for PHF and reported better active external rotation in the primary RSA group because of better consolidation of the tuberosities. However, we did not observe statistically significant differences in the rate of tuberosity consolidation between the two groups, which may explain why there were no differences in ROM. The use of an implant with a 135-degree and lateralized stem may contribute to achieving adequate ROM, regardless of tuberosity healing.

The difference in CMS, but not in ROM, could be attributed to the fact that this instrument does not include tasks involving external rotation with the arm at the side. This limitation, present in commonly used outcome scale scores, has been previously reported [25].

Subgroup analysis also revealed similar clinical outcomes in patients with prior three- or four-part fractures, both in patients who underwent acute and salvage RSA following failed osteosynthesis. In the salvage group, the primary reason for surgery was AVN in patients initially treated with ORIF. In patients who underwent HA, the main cause of revision was the lack of union of the tuberosities in three patients and malunion in one patient. Only one case of aseptic loosening occurred. Owing to the limited number of patients in the salvage RSA group, it was not feasible to perform a subgroup analysis for each cause of revision.

The choice of primary RSA for PHFs remains a topic of controversy. The literature supports the use of RSA for acute, displaced PHFs [4,5], but there have been few reports on the effect of timing on RSA outcomes. While good outcomes are generally observed with both primary and salvage RSA, there is a need to enhance our ability to select patients who would benefit from acute RSA. Panagopoulos et al. reported that the time from injury to pain-free function was significantly shorter in the primary RSA group [18]. Our study revealed similar results, where the VAS pain score was significantly lower in the acute group. This consideration is essential because despite good results following salvage RSA, we must also consider the time and healthcare costs for each patient.

Fewer complications have been reported in previous studies of primary RSA [3,18]. Previous surgical procedures have been shown to be an independent factor contributing to poor outcomes of RSA after PHF [26,27], which was not shown in our study. Additionally, some authors highlight differences in outcomes on the basis of the cause that leads to the indication for RSA, showing superior results for primary RSA and malunions compared with failed ORIF or HA [28-30].

Our study has several limitations. This was a retrospective analysis with all the inherent limitations. The limited number of patients in both groups and the inability to compare them in a subgroup analysis led to increased heterogeneity in the salvage RSA group. Although all patients provided outcome scores at a minimum of one year, the range of the most recent physical examination data and radiographic data was from earlier time points. Another limitation of the study is that the ROM and radiographic assessment were assessed by more than one observer. 

## Conclusions

While both primary and secondary RSA for the treatment of PHFs can yield successful clinical outcomes, primary RSA results in better functional scores. We did not observe any differences in other outcomes, including external rotation. This study can be used to help counsel patients when discussing the options and expectations of treatment for acute PHFs and the usefulness of RSA as a salvage option. 
